# Metabolic Regulation of Regulatory T Cell Development and Function

**DOI:** 10.3389/fimmu.2014.00590

**Published:** 2014-11-18

**Authors:** David John Coe, Madhav Kishore, Federica Marelli-Berg

**Affiliations:** ^1^Department of Biochemical Pharmacology, William Harvey Research Institute, Queen Mary University, London, UK

**Keywords:** metabolism, regulatory T cells, T cell differentiation, T cell function, mTOR pathway

## Abstract

It is now well established that the effector T cell (T_eff_) response is regulated by a series of metabolic switches. Quiescent T cells predominantly require adenosine triphosphate-generating processes, whereas proliferating T_eff_ require high metabolic flux through growth-promoting pathways, such as glycolysis. Pathways that control metabolism and immune cell function are intimately linked, and changes in cell metabolism at both the cell and system levels have been shown to enhance or suppress specific T cell effector functions. Furthermore, functionally distinct T cell subsets require distinct energetic and biosynthetic pathways to support their specific functional needs. In particular, naturally occurring regulatory T cells (T_reg_) are characterized by a unique metabolic signature distinct to that of conventional T_eff_ cells. We here briefly review the signaling pathways that control T_reg_ metabolism and how this metabolic phenotype integrates their differentiation and function. Ultimately, these metabolic features may provide new opportunities for the therapeutic modulation of unwanted immune responses.

## Metabolic Features of Regulatory T Cells

T cell differentiation and fate are orchestrated by signaling events involving the T cell receptor (TCR), co-stimulatory or co-inhibitory receptor stimulation, and cytokines. In addition, a variety of other environmental factors can also contribute to this decision. T cells switch between highly proliferative states (i.e., developing thymocytes and activated proliferating T cells) and quiescent states (i.e., naive, memory, and anergic T cells), characterized by the activation of different intracellular metabolic pathways (1). T cells use glucose as their primary fuel source for generation of adenosine triphosphate (ATP) and it is necessary for cell survival, growth, activation, proliferation, and cytokine production (2, 3).

T cell receptor stimulation is accompanied by signals from growth factors and cytokines such as interleukin (IL)-2 or IL-7, and co-stimulatory molecules, such as CD28, which lead to an increase in glucose uptake and glycolysis through induction of phosphoinositide-3-kinase (PI3K)-dependent activation of Akt (4). Akt induces glucose metabolism by facilitating glucose uptake via the upregulation of glucose transporter 1 (Glut1) on the T cell membrane (5). Failure of T cells to up-regulate glucose metabolism results in decreased cytokine production, proliferation, and ultimately to apoptosis (6–8) or anergy (9). An increase in the rate of protein synthesis also occurs following T cell activation and is regulated via Akt, which controls the activation of the mammalian target of rapamycin (mTOR), which is a key regulator of protein synthesis in T cells (10, 11).

Naturally occurring regulatory T cells (T_reg_), defined as CD4^+^CD25^+^Foxp3^+^ T cells, play a non-redundant role in the maintenance of physiological tolerance to self-antigens and prevention of autoimmune responses (12, 13). T_reg_ generation in the thymus is promoted by recognition of self-peptides with intermediate affinity (14). T_reg_ cells are characterized by a specific metabolic signature regulating their responsiveness to antigenic stimulations when compared to other CD4^+^ T cell subsets (15–18). Specifically, Th1, Th2, and Th17 cells express high surface levels of Glut1 and are highly glycolytic. T_reg_, in contrast, express low levels of Glut1 and have high lipid oxidation rates *in vitro* (19). Furthermore, blocking glycolysis promotes T_reg_ cell generation through the transcription factor hypoxia-inducible factor 1α (HIF1α), whose induction required mTOR pathway activation (20). In turn, HIF1α enhances Th17 development through direct transcriptional activation of RORγt, and concurrently, it attenuates T_reg_ development, by binding FoxP3 and targeting it for proteasomal degradation.

**Figure 1:**
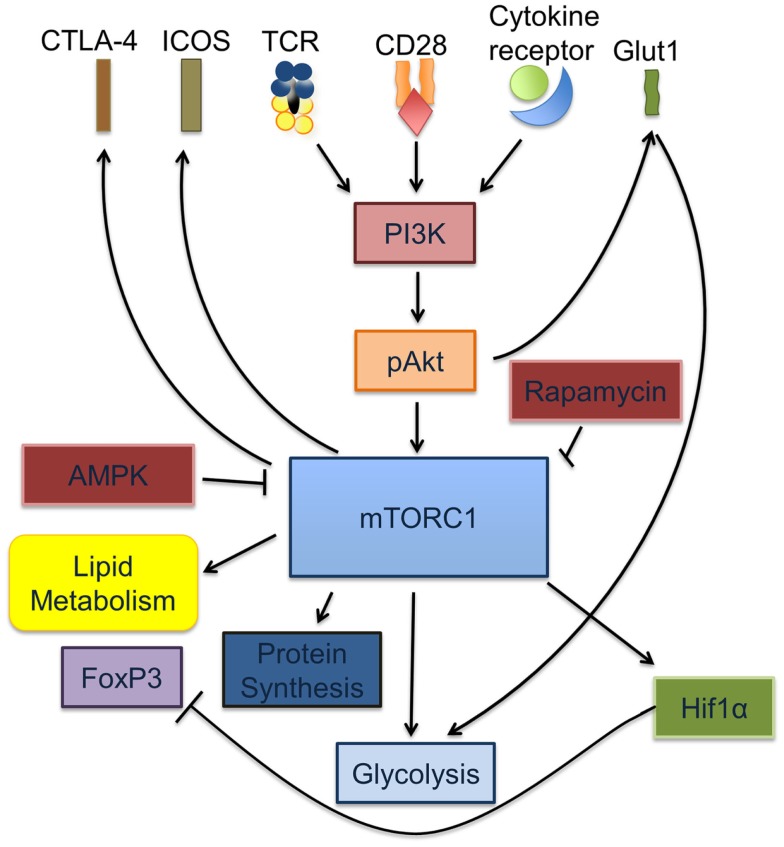
**mTORC1 is a key regulator of T_reg_ and T_eff_ activation and differentiation**. Signaling through the TCR is accompanied by cytokine signals from IL-2, IL-7, and leptin as well as co-stimulation via CD28. Together, these signals cause increased glycolysis through the induction of PI3K-dependent activation of Akt, which in turn increases glucose transport via the upregulation of Glut1 on the T cell surface. Activated pAkt increases the rate of protein synthesis via activation of mTORC1. Activated mTORC1 activates HIF1α, regulates lipid metabolism and glycolysis.

Collectively, these observations underscore the key role of metabolic cues and regulatory pathways in defining T cell differentiation and function (Figure 1).

## mTOR Inhibition and T_reg_ Differentiation

The mTOR is a key regulator of T cell metabolism, that serves to integrate nutrient sensing pathways with signaling pathways involved in differentiation, growth, survival, and proliferation (21). TCR and co-stimulatory signals along with cytokines tweak the mTOR pathway via the upstream PI3K/Akt signaling networks to match the energy requirements associated with T cell activation (22, 23). Conventional CD4^+^ and CD8^+^ T cells, upon stimulation, utilize the mTOR pathway to meet the increased metabolic demands of T cell activation by switching from primarily oxidative phosphorylation, seen in resting T cells, toward a state of enhanced aerobic glycolysis, a phenomenon popularly described as the Warburg effect (3, 24, 25). The importance of this phenomenon in determining T cell fate was first noticed using the selective inhibitor of mTOR, rapamycin, which prevented the generation of T_eff_ responses and promoted the generation of T_reg_ cells (9, 26–28). Additionally, T cell-specific mTOR knockouts were shown to have poor T_eff_ responses and defaulted toward a more T_reg_ phenotype (29). These studies not only revealed the importance of mTOR as a critical regulator in the differentiation of T_reg_, but also highlighted the importance of the metabolic pathways that predominate within functionally different T cell subsets.

Consistent with the above findings, T_reg_ display higher levels of AMP kinase activity and preferential lipid oxidation for their energy requirements (19). The AMP-activated kinase acts as a sensor of the AMP/ATP ratio, which is increased during hypoxia and inhibits mTOR kinase to promote mitochondrial oxidative metabolism rather than glycolysis (30, 31). Interestingly, activation of AMP kinase via Metformin, a drug used to treat diabetes mellitus, increased the T_reg_ population in the CD4^+^ T cell compartment in an *in vivo* murine model of asthma (19). In this study, mice sensitized by aerosol to ovalbumin in the presence of metformin, and challenged 21 days later showed an increase in the frequency and number of CD4^+^Foxp3^+^ T cells in the draining lymph nodes as compared to mice immunized in the absence of metformin. However, no change in airway responsiveness was noted even though there were fewer lymphocytes recovered in the bronchial alveolar lavage in the metformin treated animals. Additionally, inhibition of mitochondrial lipid uptake and oxidation pathways by Etomoxir, an inhibitor that prevents long chain fatty acid uptake to the inner mitochondrial membrane for beta oxidation, abrogated the generation of T_reg_ without altering T_eff_ differentiation (19). Furthermore, T_reg_ were shown to express lower levels of the glucose transporter Glut1 as compared to T_eff_, and transgenic CD4^+^ T cells overexpressing Glut1 were shown to develop fewer T_reg_. Overall, these studies indicate that fatty acid oxidation is the dominant metabolic process utilized for the generation of energy in T_reg_.

## mTOR and T_reg_ Function

While inhibition of mTOR enhances T_reg_ generation during an immune response, mTOR activity is known to be required to maintain their suppressive capabilities. In this section, we review recent findings that investigated this apparent dichotomy in the function of mTOR in T_reg_ biology. mTOR exists as two structurally distinct complexes (mTORC1 and mTORC2). Both complexes localize within different subcellular compartments and have different functions in the cell; rapamycin-sensitive mTORC1 forms the fundamental nutrient sensing complex that is activated by Akt kinase downstream of PI3K signaling induction (via the TCR, co-stimulatory receptors, and cytokines) whereas the rapamycin-insensitive mTORC2 controls spatial aspects of cell growth through activation of cytoskeletal components (32, 33). The mTORC2 complex also, in turn, activates the kinase Akt (34, 35). Thus, Akt lies both upstream and downstream of mTOR. In mice, CD4^+^ T cells lacking both mTORC1 and mTORC2 complexes fail to differentiate into any T_eff_ lineage (Th1, Th2, or Th17) and instead differentiate toward the T_reg_ cell phenotype, consistent with the CD4^+^ population of mTOR null mice (36). However, recent findings by Hu Zheng et al. indicate a crucial role of the mTORC1 complex to the suppressive activity of T_reg_ (29). Indeed, mTORC1 activity was shown to be higher in T_reg_ than naive T cells under steady state conditions. Impairment of the mTORC1 pathway in T_reg_ via selective genetic deletion of Raptor, an obligatory component of mTORC1, in the CD4^+^ FOXP3^+^ compartment, led to the early onset of a fatal autoimmune disease in mice (29). Moreover, the disease mimicked the autoimmune disease seen in Scurfy mice that bear a loss-of-function mutation in the FoxP3 transcription factor, indicating impaired T_reg_ function. Mechanistically, the mTORC1 pathway in T_reg_ was shown to be necessary to initiate the upregulation of surface CTLA-4 and ICOS, key intrinsic receptors for T_reg_-mediated suppression. In addition, mTORC1 was shown to induce cholesterol and lipid metabolism as well as proliferation in the T_reg_ population (29). Finally, recent investigations have revealed a non-redundant role of mTORC1 in mitochondrial metabolism (37). Collectively, these investigations imply a differential use of mTOR in T_reg_ as compared to conventional effector cells.

## A Model of T_reg_ Differentiation Based on mTOR Activation

**Figure 2:**
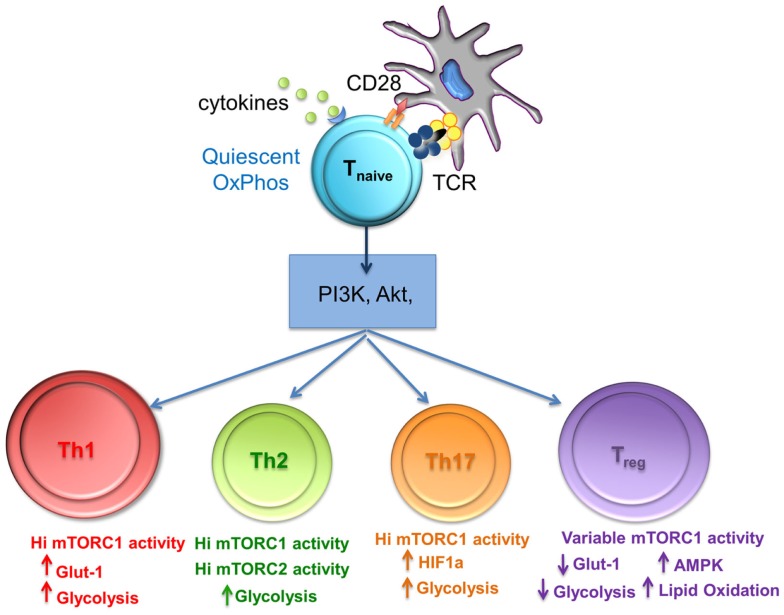
**The level of mTORC1 activity and glycolysis is important for differentiation of T cell subsets**. mTORC1 integrates nutrient sensing and signaling pathways to match the energy requirements of activated T cells. Th1, Th2, and Th17 cells require high levels of glycolysis that is mediated by high mTORC1 activity, whereas T_reg_ differentiation requires variable mTorc activity, reduced glycolysis, and lipid oxidation.

From the aforementioned studies, it is clear that the metabolic cues from the environment and subsequent mTOR activity play a key role in T_reg_ differentiation. Powell et al. have proposed a model of T_reg_ differentiation based on mTOR activity that mimics that seen in conventional T cell differentiation. Briefly, naïve T cells, receiving strong mTOR activation upon antigen recognition (through environmental cues, TCR, cytokine, and co-stimulatory stimulation), differentiated into short-lived T_eff_ cells exhibiting high glycolytic activity, while those receiving weak mTOR activation developed into long-lived memory T cells dependent on oxidative phosphorylation to meet their energy needs (38). One can suggest that the high level of mTOR activity in T_eff_ cells would be necessary to sustain higher demand for energy via glycolytic pathways while the opposite would hold true for quiescent memory T cells. A similar model can be applied to induced T_reg_ where naïve T cells in the presence of TGF-β receiving either high or low mTOR activating signals could result in the differentiation of “effector” and “memory” Foxp3^+^ T_reg_ respectively_._ As such, CD4^+^ Foxp3^+^ T cells that traffic to activating lymph nodes and become robustly stimulated (mTOR^hi^) generate short-lived “effector” T_reg_. These effector T_reg_ would then home to the tissues and control immune responses. This model can explain why T cells stimulated *in vitro* with high doses of peptide in the presence of exogenous TGF-β develop into T_reg_. These mTOR^hi^ T_reg_ exhibit high glycolytic activity similar to that of conventional T_eff_ cells (Figure 2). Consequently, this model can also be applied to natural T_reg_ cells differentiation into effector or memory T_reg_ arising through associated mTOR hi or low activity upon antigen recognition (38).

## Oscillating mTOR Activity Promotes Proliferation in T_reg_

A hallmark feature of T_reg_ cells is their ability to proliferate abundantly *in vivo* while remaining anergic and poorly proliferative *in vitro* (39, 40). This anergic *in vitro* state was shown to be reversible via activation in the presence of supra-physiologic concentrations of IL-2 (41). In addition, short-term treatment of T_reg_ with rapamycin preceding activation in the presence of supra-physiologic quantities of IL-2 was shown to promote proliferation *in vitro* at much higher levels than those induced by IL-2 alone. This posed a conundrum as to how two signals having opposite effects on mTOR activity can converge to enhance proliferation of T_reg_. To explain this phenomenon, a model was put forward (18), which postulates that mTOR activity in T_reg_ is highly dynamic, oscillating between low and high activation states. As mentioned before, mTOR activity in T_reg_ was shown to be higher at resting states when compared to naïve T_eff_. According to this model, the intermittent reduction in mTOR signaling followed by its enhanced activation by means of TCR triggering and IL-2 stimulation promotes T_reg_ proliferation. However, T_reg_ requirement for down-regulation of mTOR signaling was shown to be short-lived as protracted incubation with rapamycin ablated their proliferation. This model also identified the adipocyte hormone leptin as a key signal that regulates mTOR activity *in vivo*, promoting T_reg_ proliferation. Within the immune system, leptin has been seen to activate pro-inflammatory cells while diminished leptin levels can lead to immunosuppression (42). Leptin produced by T_reg_ cells was shown to contribute to the activation of the mTOR pathway in an autocrine manner. Other mechanisms through which mTOR activity is maintained in its oscillating state to overcome their hypo-responsiveness and enter the cell cycle continue to be investigated.

## Metabolic Regulation of T_reg_ and Th17 Differentiation

Interleukin-17 (Th17) producing and induced regulatory T cells (iT_reg_) differentiate from naïve CD4^+^ T cells and mediate diverse and often opposing effects in lymphoid and peripheral tissues. Under the influence of TGFβ and IL-2, naive T cells are induced to express the transcription factor FoxP3, and differentiate into tissue-resident iT_reg_, which support a suppressive environment. However, in the presence of IL-6, naive T cells stimulated with TGFβ express the transcription factors STAT3 and Rorγt, secrete IL-17, and produce an inflammatory environment.

It has recently emerged that metabolic factors can modulate the balance of Th17 and iT_reg_ cells resulting in inflammation or actively maintained tolerance.

Commitment to the Th17 lineage, like other T_eff_, requires increased mTORC1 activity to sustain differentiation and function. As the presence of TGFβ is required for the development of both Th17 and T_reg_ cell subsets, the relative differentiation of each cell type can be influenced by the level of mTORC1 activation. This interconnectivity is especially significant because of the opposing functions of the two cells types. The metabolic regulation and influence on the Th17:T_reg_ ratio has been articulately reviewed by Barbi, Pardoll, and Fan-Pan (43) and so is briefly summarized here.

The activation of mTOR, and the subsequent switch to aerobic glycolysis, is essential for Th17 development; IL-1 enhances Th17 cell differentiation and proliferation via mTOR activation (44) whereas mTOR inhibition prevents Th17 differentiation (45, 46) and ameliorates Th17-dependent symptoms in a murine EAE model (47). Concomitantly, in these experiments, mTOR inactivation increases T_reg_ cell numbers and function and sensitizes T_reg_ to TGFβ (45, 48).

As well as mTOR, hypoxia-inducible factor (HIF1α), a transcription factor activated during inflammation and in response to low oxygen levels, is a critical regulator of metabolism. In T cells, HIF1α plays a role in inducing aerobic glycolysis even in the presence of plentiful oxygen (49). Elevated glycolysis in Th17 cells is dependent on HIF1α, and indeed, the transcription factor is essential for their differentiation and function (20). HIF1α activation, under aerobic conditions, is modulated by mTORC1 and therefore the concerted actions of HIF1α and mTORC1 preferentially guide Th17 cell development and effector functions. Furthermore, HIF1α directly binds to FoxP3 and targets it for proteosomal degradation while also increasing the transcription of the Th17-related transcription factor Rorγt. Mice with HIF1α-deficient T cells are resistant to Th17-dependent EAE with a response that is characterized by a decrease in Th17 cells and an increase in T_reg_ cells (50). Thus, HIF1α and mTOR represent important mediators of the Th17:T_reg_ balance in hypoxic and inflamed tissues, and as such are potentially important targets for clinical interventions.

## Visceral Adipose Tissue-Associated T_reg_

Metabolic stress is also known to influence the development of T_reg_, and specifically to affect adipose-tissue-resident T_reg_ cells. This population of T_reg_ produces high levels of IL-10 and is characterized by the expression of GATA3, CCR2, KLRG1, and lack of CD103 expression (51). Visceral adipose tissue (VAT) T_reg_ are thought to be important for the maintenance of responsiveness to insulin, by regulating adipokine release. In obese humans and mice, VAT T_reg_ are progressively replaced by a pro-inflammatory T_eff_ cell infiltrate, which accumulates in adipose tissue and produces cytokines that causes systemic low grade chronic inflammation (52–54), subsequently leading to insulin resistance and other obesity-related morbidities. VAT resident T_reg_ negatively regulate inflammation and represent a tissue specific T_reg_ population that express a distinct T cell repertoire (52) and a unique transcription factor, peroxisome-proliferator-activated receptor γ (PPARγ) (51). Obesity in mice and humans causes a reduction in VAT-associated T_reg_ differentiation (55). Moreover, removal of VAT resident T_reg_ by conditional knock-out of PPARγ, or activation, by treatment with pioglitazone, modulates levels of inflammatory cell subsets and insulin sensitivity (51). Leptin, a class I cytokine, is produced in higher amounts by adipocytes in obese individuals and inhibits rapamycin-induced proliferation of T_reg_ via increased activation of mTORC1 (18, 56). Leptin, secreted in the VAT, therefore, represents a potential regulator of the function of adipose-tissue-resident T_reg_. In contrast to leptin, adiponectin, an anti-inflammatory adipokine, retains insulin-sensitizing properties and negatively correlates with body mass index while positively correlating with T_reg_ cell representation in VAT (57).

## Amino Acid Concentration Regulates T_reg_ Differentiation and Function

Regulatory T cell differentiation and function are also controlled by the availability of amino acids in the local milieu. The essential amino acids arginine, glutamine, and tryptophan are essential for T cell activation (58–61) and their depletion from the local microenvironment results in T_reg_ generation. For example, Tryptophan is catabolized by indoleamine 2,3-dioxygenase (IDO) and tryptophan 2,3-dioxygenase (TDO), which are present on many suppressive cell types including regulatory dendritic cells (DC) and some tumors. Low concentrations of tryptophan inhibits T cell growth but enhances T_reg_ generation (62–64) through an mTOR-dependent mechanism (65). The depletion of arginine by arginase (ARG1) and nitric oxide synthase (iNOS) also inhibits T cell activation via mTor inhibition. ARG1, iNOS, and IDO can be induced by T_reg_ in actively tolerant skin grafts *in vivo* (66) providing a feed-back loop by which T_reg_ can influence amino acid availability via autocrine mTOR activation and subsequently control T_eff_ activation and function. The influence of amino acid metabolism on T_reg_ differentiation and function has been reviewed elsewhere (67).

## Therapeutic Implications

The metabolic pathways influencing T_reg_ differentiation and function are amenable for modulation in therapeutic settings, thus providing the clinician with potentially valuable tools in the fight against immune-mediated diseases. As the mechanisms by which Rapamycin affects T_reg_ function are elucidated, more areas of clinical intervention will be opened for this FDA approved, well tolerated, and bio-available drug. To this end, it has recently been demonstrated by Makki et al. (68) that the metabolic effects of Rapamycin can protect against insulin resistance, increase energy expenditure, and reduce weight gain in diet-dependent obese mice. These phenotypic effects correlate with an increase of T_reg_ and myeloid derived suppressor cells in the adipose tissue (68). These finding will certainly fuel the debate over the use of Rapamycin beyond organ transplantation.

Proglisterone, which is currently licensed as a drug for the treatment of Type II diabetes, provides another potential target to modulate T_reg_ metabolism. Proglisterone is known to stimulate PPARγ and when used to treat mice fed a high fat diet, it restores the number and function of visceral adipose specific T_reg_ and this effect appears to be PPARγ specific (51). Therefore, Proglisterone can potentially target pathologies related to VAT T_reg_ with no bystander effects on other T_reg_ populations. The regulation of accumulation and function of PPARγ^+^ T_reg_ by leptin and adiponectin represents a potentially valuable therapeutic pathway that may, in the future, be targeted in order to regulate obesity-related pathologies. Moreover, the role of leptin and T_reg_ in the progression of obesity-related diabetes is yet to be fully elucidated and may provide even more targets for future drug research.

On another note, and potentially related to T_reg_ dependence on fatty acid metabolism, short-chain fatty acids (scFA), of bacterial origin (i.e., propionate, butyrate, and acetate), can restore the T_reg_ compartment in the gut of germ-free mice that had been treated with irradiation or antibiotics. This re-population is partially dependent on the expression of free fatty acid receptor 2 (FFAR2) on colonic T_reg_, which physiologically express higher levels of FFAR2 than other T_reg_ sub-populations. This observation opens up the tantalizing possibility that colonic T_reg_ may be specifically targeted, in clinical settings, using synthetic scFA to treat gut-related problems in immunocompromised individuals (69).

## Concluding Remarks and Perspectives

The recent ground breaking research in how metabolism effects T_reg_ biology has provided the scientific and medical community with a plethora of novel mechanistic insights that will inevitably lead to a better understanding of disease and a host of therapeutic targets. However, we still need to understand how the varying tissue-specific transcription factors found in T_reg_ sub-populations are influenced by their environment, external and internal metabolic factors. The expression of PPAR-γ by VAT T_reg_ suggests that the metabolic environment can influence the expression of transcription factors not only in resident cells but also in new migrants to the tissue. A future challenge will involve extending this concept to establish whether the metabolic microenvironment, which characterizes different tissues, can determine the balance of regulation versus inflammation *in situ*. If true, this possibility will pave the way for organ-selective immune-metabolic therapy.

## Conflict of Interest Statement

The authors declare that the research was conducted in the absence of any commercial or financial relationships that could be construed as a potential conflict of interest.
